# Morphometric variation in wolves and golden jackal in India (Mammalia, Carnivora)

**DOI:** 10.3897/BDJ.9.e67677

**Published:** 2021-08-27

**Authors:** Yellapu Srinivas, Yadvendradev Jhala

**Affiliations:** 1 Wildlife Institute of India, Dehradun, India Wildlife Institute of India Dehradun India

**Keywords:** canids, cranium, hair, forensics, Principal Component Analysis, taxonomy

## Abstract

Species of *Canis* (Carnivora, Canidae) have similar morphology and distinguishing sympatric species is challenging. We present data on morphometry of skull, body and hair of three wild *Canis* species that occur in India, which include two wolves (Indian wolf, *Canislupuspallipes*; and Himalayan wolf, *Canishimalayensis*) and the golden jackal (*Canisaureus*). A total of 20 cranial and six body measurements and microscopic characteristics of guard hair were analysed, using multivariate ordination to differentiate between species. Cranial measures of the Himalayan wolves were found to be the largest followed by Indian wolves and golden jackals. However, many measures overlapped amongst the three species. Two Principal Components each, for body measures and cranial measures, explained 86 and 91% of the variation in the data, respectively. These Components discriminated the two wolves from golden jackals, but could not distinguish between wolves. Hair medullary patterns were simple and wide type, whereas hair cuticular patterns showed crenate scale margins, near scale distance and irregular wavey scale patterns for all *Canis* taxa and were not useful to distinguish species. Data reported in this study further contribute to the existing global data on wild canids for a holistic understanding of the variation within the genus and show that distinguishing between all sympatric species from morphology alone may not be possible.

## Introduction

**Figure 1.**
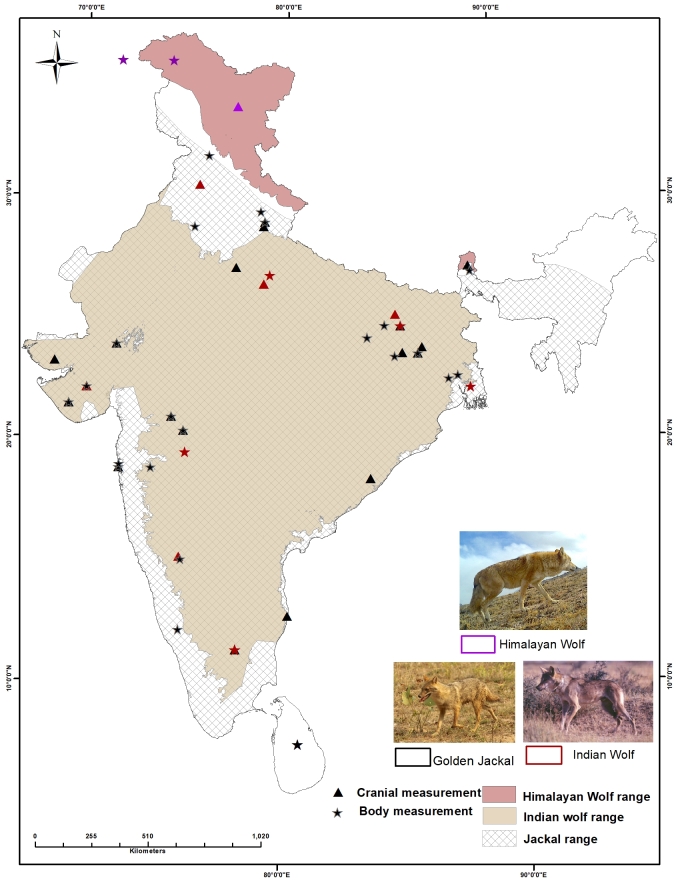
Distribution range of wild *Canis* species in India along with sampling locations. The golden jackal distribution was obtained from the IUCN species database (http://maps.iucnredlist.org/map.html?id=3744, accessed 16 June 2021), while the Indian and Himalayan wolf range is depicted from locations and maps available in Aggarwal et al. 2003, Jhala 2003 and Sharma et al. 2004.

Three wild *Canis* species occur in India: the Indian wolf (*Canislupuspallipes*), the Himalayan wolf (*Canishimalayensis*) and the golden jackal (*Canisaureus*; Fig. 1). The range of the two wolves differs significantly since the Himalayan wolf occurs only in the high altitude trans-Himalayas of India, Nepal, Bhutan, Tibet and, possibly, Pakistan, while the Indian wolf is restricted to the plains of peninsular India and Pakistan (Jhala 2003). The range of the golden jackal overlaps the entire range of the Indian wolf, but co-occurrence between golden jackals and Himalayan wolves is extremely rare (Jhala and Moehlman 2004). Another wild canid, the Asiatic wild dog (*Cuonalpinus*), also known as the dhole, is often found in sympatry with all the three *Canis* species. IUCN lists grey wolves and golden jackals as least concern (Boitani et al. 2020, Hoffmann et al. 2020). However, regionally, both wolves in India are considered endangered and protected under Schedule I of the Indian Wildlife (Protection) Act, 1972 and Appendix 1 of the Convention on International trade in Endangered Species of Wild Fauna and Flora (CITES). Golden jackals are also protected under Schedule II (part II) of the Indian Wildlife (Protection) Act, 1972. The Act provides absolute protection for Schedule I and Schedule II species and offences under these are prescribed the highest penalties.

Wolves in India (Indian and Himalayan) are considered ancient and distinct from the wolf-dog clade (Aggarwal et al. 2003, Sharma et al. 2004). Yumnam et al. (2015) highlighted that the Indian golden jackals were genetically diverse with the presence of unique haplotypes and were possibly ancestral to all western golden jackal populations. Across most of their distribution, the majority of which is outside of protected areas, Indian wolves and jackals have either disappeared or undergone drastic declines due to anthropogenic impacts (Pillay et al. 2011). Poisoning of wolves, poaching for pelts in the Himalayas, killing jackals for religious practices (jackal horns and tails), and fragmentation of populations by linear infrastructure were considered as the major threats to the survival of wild *Canis* species in India (Chawla et al. 2020, Jhala 2003). Moreover, an increasing population of free-ranging dogs poses a serious threat to the survival of wild *Canis* species by hybridizing and swamping wild gene pools (Vilà and Wayne 1999), spreading infectious diseases (Vanak and Gompper 2009), and competing with wild canids for resources (Jhala and Giles 1991).

Identifying individual animals and populations to the species level is important for conservation management and policy formulation (Frankham et al. 2002), as well as for wildlife forensics (Bellis et al. 2003). Segregation of wolf species and subspecies has been done, based on geographical separation, morphological differences, such as pelt colour, body size, skull and skeletal measures, and behaviour (Wozencraft 2005). Globally, quantitative interspecific and intraspecific variations within *Canis* species show large overlaps between geographic localities and sexes, especially in craniometrical characteristics (Milenković et al. 2010, Okarma and Buchalczyk 1993, Stoyanov 2020, Nowak and Federoff 2002). In India, to date, no major study has been published on morphometric variations within and between the Indian wolves, Himalayan wolves and golden jackals. In this study, we report morphometric variations of the skull, body, and hair amongst wild *Canis* species in India and assess the reliability of these measures to discriminate amongst the three taxa.

## Sample collection and methodology

We measured samples from the historical collection of the Bombay Natural History Society (BNHS), from the Wildlife Institute of India (WII), from individuals captured for radio-telemetry study, from road kills and those provided to us by wildlife authorities for forensic investigations. Only adult samples without differentiating between males and females of each species were used. Samples that were of uncertain origin or ambiguous (hybrids) in nature were not included in this study. All measurements were recorded by the authors. All live animals were captured after obtaining permissions under the Wildlife (Protection) Act, 1972 from the Chief Wildlife Warden.

### Cranial and external body characters

Skulls of Indian wolves (n = 12), Himalayan wolves (n = 4), and Golden Jackals (n = 33) were sampled from the mammal collections of BNHS and WII. Adults were identified, based on the zygomatic breadth and fused spheno-occipital sutures (Gipson et al. 2000). Skulls with no external damage were included in the analyses. A total of 20 craniometric measurements (Table 1, Suppl. material 1) were recorded for all three species following Onar et al. (2005), Milenković et al. (2010). All cranial measurements were recorded using a digital caliper with an accuracy of 0.01 mm. Skin samples of Indian wolves (n = 11), Himalayan wolves (n = 4) and golden jackals (n = 52) were also measured from BNHS and live animals measured during a radio-collaring exercise. Body measurements were also recorded from skins and live animals.(Table 1, Suppl. material 2).

### Hair morphology

Reference guard hair samples from the dorsal body region of Indian wolf, Himalayan wolf and jackal were obtained from the repository skin samples of WII. A minimum of 10 hairs were taken from each sample for microscopic examination of cuticular and medullary patterns. Hair samples were thoroughly washed with hydrogen peroxide and xylene to clear dirt and opacity. Cuticular impressions were prepared on a thin film of saturated gelatine solution (Koppikar and Sabnis 1976). Cuticular and medullary patterns of each species were then captured using a Leica F 300 (Leica Microsystems, Germany) on microscopic glass slides under 400× magnification following standard methodology as described in Brunner and Coman (1974), Singh et al. (2020). Cuticular patterns, medullary patterns along the length of the hair shaft and medullary margins along the shield regions were considered for hair morphological analysis.

### Statistical analyses

Mean values along with standard errors for skull and body measures were computed. Principal Component Analysis (PCA) was carried out on log-transformed morphometric data (external body measures and skull) to reduce dimensionality and collinearity amongst variables (Jolliffe 2002). Subsequently, individuals were segregated into clusters, based on ordination of their principal component (PC) scores and 95% confidence ellipses generated for each species. PCA analysis was carried out using packages ggplot2, grid and grid extra in R software 3.0.1 (RCoreTeam 2013).

## Results

### Variation in cranial and external body characters

**Figure 2.**
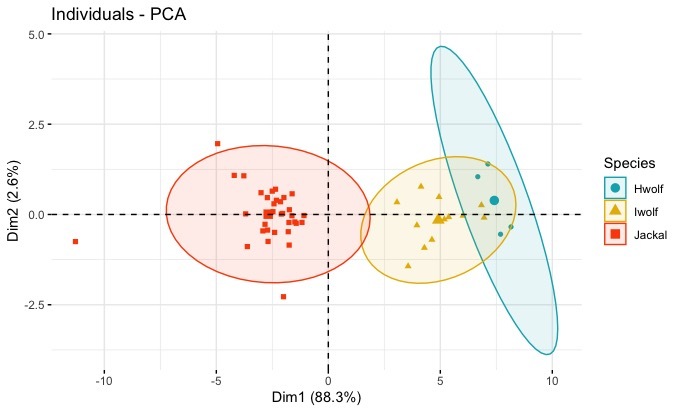
Segregation of golden jackal, Indian wolf and Himalayan wolf, based on two principal components of cranial measurements. Blue, yellow and red colours represent Himalayan wolves, Indian peninsular wolves and golden jackals, respectively. The first and second axes of the PCA explained by 88.3% and 2.6% of the variation observed in the data.

Based on the variables used in this study, PCA showed clear discrimination between skulls of wolves and golden jackals and with an overlap of the 95% ellipses of the two wolves (Fig. 2). Himalayan wolves had the largest skull measurements and golden jackals had the smallest. Except for M2 length, mean values of all other variables were found to be larger in the Himalayan wolf in comparison to the Indian wolf (Table 1). The first and second principal components are explained by 88.3% and 2.6% of the variation observed in the data (Suppl. material 3). Factor loadings of all variables on PC1 were almost equal, while PC2 had maximum loading from variables associated with the size of the mandible (Fig. 2; Suppl. material 3) (Fig. 2).

**Figure 3.**
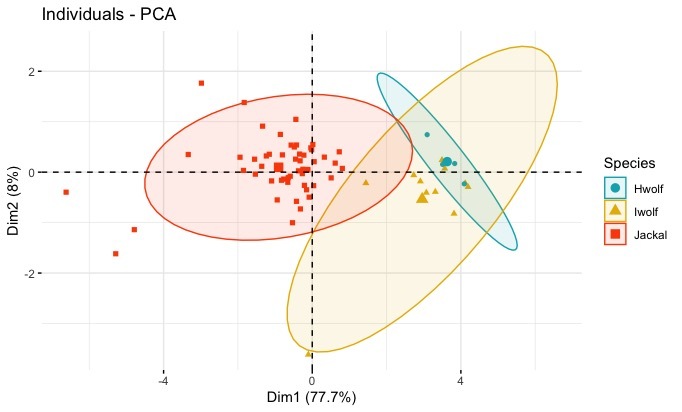
Segregation of golden jackal, Indian wolf and Himalayan wolf, based on two principal components of external morphological measurements. Blue, yellow and red colours represent Himalayan wolves, Indian wolves and golden jackals, respectively. The first and second axes of the PCA explained by 77.7% and 8.0% of the variation observed in the data.

Body measures differentiated wolves from golden jackals, with wolves having larger mean values in comparison to golden jackals. The first two principal components explained 77.7% and 8.0% of the variability in the data, respectively (Suppl. material 4). PC1 loadings of all body measures were comparable and positive, while PC2 had maximum loading from the length of the tail (Suppl. material 4). Ordination on the two PC axes of body morphology measures showed clear discrimination between wolves and jackals (Fig. 3). However, species of wolves overlapped substantially and discrimination between them was not possible.

### Analysis of hair morphology

**Figure 4.**
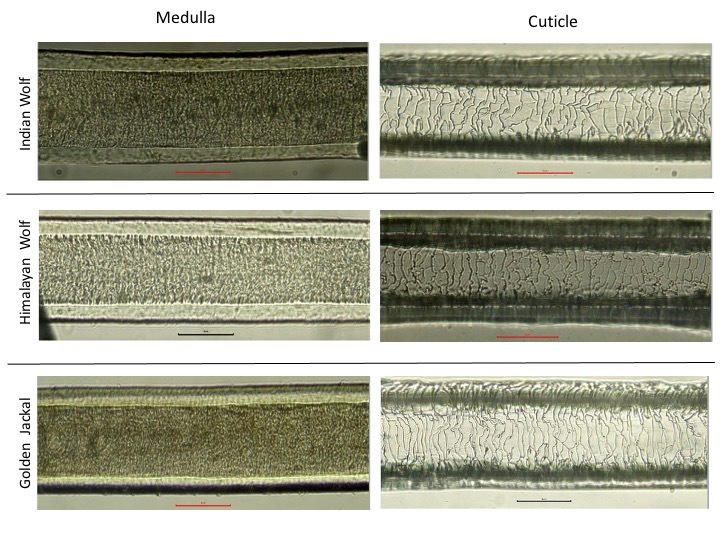
Cuticular and medullary structures of guard hair of Indian wolf, Himalayan wolf and golden jackals.

Based on the analysis of hair morphology, major variations were not observed amongst the Indian wolves and jackals that can be used for species identification (Fig. 4). Cuticular pattern of hair from all the Indian wolves and golden jackals showed a crenate margin, near scale distance, and irregular wave scale patterns (Fig. 4). Medullary characteristics across all the three species were also similar with simple and wide medulla type. Medulla thickness was observed to vary between species, with the thickest medulla observed in Indian wolf (0.075 mm, SE:0.001) and thinnest in Himalayan wolf (0.054 mm, SE:0.001). Percentage medulla (ratio of medullary thickness to the total thickness of the hair) ranged between 70.2% (SE:0.47) and 81.4% (SE:0.7) in the Indian wolf and the Himalayan wolf, respectively (Table 2).

## Discussion

Despite advances in molecular taxonomy, morphology still plays an important role in phylogenetic studies, distinguishing individuals and populations for conservation management and for forensic applications (Rutledge et al. 2012, Hinton and Chamberlain 2014). We believe that the information we present here will address the void in morphological measures available for Indian *Canis* species and assist in more comprehensive and holistic studies of canids globally. Variation in cranial measures within the same species collected across India (Suppl. material 1) was not significant. Though our sample coverage (Fig. 1) was across the range for Indian wolves and jackals, the sample sizes were small and inadequate to address geographical variation within species. Cranial measures of Himalayan wolves were largest followed by Indian wolves and golden jackals. These results are in agreement with the findings of Okarma and Buchalczyk (1993) who reported that mountain species have larger skulls. The canids, sympatric with the Himalayan wolves, were red fox (*Vulpesvulpes*), Tibetan sand fox (*Vulpesferrilata*) and dhole (*Cuonalpinus*). The two foxes are rather small to be confused with wolves, while the dhole, which is smaller in size in comparison to the Himalayan wolf, is about the same size as the Indian wolf, but has a distinctive pelage and skull morphology different from *Canis* species (Wayne 1986). Skulls of jackals were smaller amongst the studied wild species and the values were consistent with the results of Stoyanov (2020). Craniometrical measures between males and females of the same species can be different (Okarma and Buchalczyk 1993, Milenković et al. 2010, Khosravi et al. 2012), but in this study, due to lack of information on sex, we could not carry out sex-based analysis. Further sampling with information on sex and additional samples across the species range is required to study sexual dimorphism and geographical variation within species. PCA results considering body measures also showed similar patterns to those of cranial measures amongst wild *Canis* species (Table 1, Suppl. material 1). Our results failed to discriminate between the two wolves, but could discriminate jackals from wolves by considering cranial and body measurements (Figs 2, 3).

Medulla and cuticular patterns of some Indian mammals were described by Chakraborty and De (2001), Bahuguna et al. (2010), Singh et al. (2020) who emphasised the importance of microscopic examination of hair as being of taxonomic value. Microscopic examination of guard hair revealed no clear variation in the medulla and cuticular patterns within wolves and jackals (Fig. 4). Sari and Arpacik (2018) also found similar results in the medulla and cuticular patterns of canids from Turkey. Using percentage medulla width, we were able to differentiate Himalayan wolf (having medullary width of 81.4% [SE: 0.7]) from other *Canis* species (70-75%), but failed to differentiate between Indian wolves and jackals (Table 2) . Even though Jhala and Sharma (1997) used electron-microscopic analysis of hair for forensic purposes to distinguish Indian wolf from dogs, their inference was based on qualitative assessments. Kennedy (1982) reported that wolf and coyote hair were distinguished from dogs, based on total length and colour. However, in this study, we did not find much variation within hair characteristics of wild canids. The hair characters described in this study for Indian wild *Canis* species could be useful in distinguishing them from other mammalian taxa including bovids, felids, cervids, primates and viverrids for forensic and dietary studies. (Bahuguna et al. 2010).

## Conclusions

Our data contribute to the existing global data on wild *Canis* species for a better and holistic understanding of the variation within the genus and allows for discrimination between jackals and wolves, but not between the two species of wolves. Since the ranges of the two wolves do not overlap, rarely would there be a need for distinguishing between them and morphometry would suffice to allocate *Canis* samples to species, provided they were accompanied by geographical location information.

## Supplementary Material

21E2E410-529F-5872-88D0-3D48AD69D619 10.3897/BDJ.9.e67677.suppl1 Supplementary material 1 Table S1: Data on cranial measures of Indian wolf (*Canislupuspallipes*), Himalayan wolf (*Canishimalayensis*), and golden jackal (*Canisaureus*) from India Data type Morphological data Brief description Data on cranial measures of Indian wolf (Canislupuspallipes), Himalayan wolf (Canishimalayensis) and golden jackal (Canisaureus) from India. File: oo_557820.xlsx https://binary.pensoft.net/file/557820 Yellapu Srinivas and Yadvendradev Jhala

CF1F0370-E7F9-579B-8BEF-7CE40326D5B0 10.3897/BDJ.9.e67677.suppl2 Supplementary material 2 Table S2: Data on body measures of Indian wolf (*Canislupuspallipes*), Himalayan wolf (*Canishimalayensis*) and golden jackal (*Canisaureus*) from India Data type Morphological data Brief description Data on body measures of Indian wolf (*Canislupuspallipes*), Himalayan wolf (*Canishimalayensis*) and golden jackal (*Canisaureus*) from India. File: oo_557821.xlsx https://binary.pensoft.net/file/557821 Yellapu Srinivas and Yadvendradev Jhala

BC2E4904-C245-5377-BDAA-A87228986E59 10.3897/BDJ.9.e67677.suppl3 Supplementary material 3 Table S3: Cumulative percentage of explained variance and contribution of the cranial variables to principal components of PCA Data type Morphological data Brief description Cumulative percentage of explained variance and contribution of the cranial variables to principal components of PCA. File: oo_531650.xlsx https://binary.pensoft.net/file/531650 Yellapu Srinivas and Yadvendradev Jhala

A4E8A6FE-C288-533A-855E-375FE7CFF490 10.3897/BDJ.9.e67677.suppl4 Supplementary material 4 Table S4: Cumulative percentage of explained variance and contribution of the external body variables to the principal components of PCA Data type Morphological data Brief description Cumulative percentage of explained variance and contribution of the external body variables to the principal components of PCA. File: oo_531651.xlsx https://binary.pensoft.net/file/531651 Yellapu Srinivas and Yadvendradev Jhala

## Figures and Tables

**Table 1.** Cranial (mm) and body (cm) measurements of adult golden jackals (n = 33 skull, 52 body), Indian wolves (n = 12 skull, 11 body) and Himalayan wolves (n = 4 skull, 4 body) from India.

S no.	Cranial Characters	Golden jackal (n = 33)					Indian wolf (n = 12)			Himalayan wolf (n=4)			
														Minimum	Maximum	Mean	Standard error	Minimum	Maximum	Mean	Standard error	Minimum	Maximum	Mean	Standard error
		1	Skull: Length	109.1	155.7	143.68	1.66	188	221	202.64	2.51	198.27	234	214.84	8.27
2	Palantine Length	55.54	79.86	73.79	0.85	99.12	111.1	105.72	1.28	106.78	120.43	113.51	2.9
3	Width between P4s	30.73	59.72	49.77	0.78	57.23	73.25	64.14	1.58	69.71	79.25	73.68	2.23
4	Width between upper canines	17.54	29.4	26.27	0.41	35.11	42.75	39.05	0.7	39.42	46.7	42.09	1.58
5	P4 length	10.83	17.77	16.27	0.22	18.5	22.8	21.16	0.35	22.71	24.85	23.93	0.45
6	M1 length	8.04	12.52	11.4	0.16	13.11	15.69	14.26	0.26	14.94	16.01	15.46	0.22
7	M2 length	5.12	7.29	6.39	0.07	6.61	8.84	7.91	0.17	7.01	8.45	7.78	0.3
8	Canine length	4.58	8.11	7.17	0.15	10.09	12.07	11.31	0.19	14.11	14.37	14.21	0.06
9	Canine width	2.94	5.06	4.44	0.08	5.83	7.25	6.66	0.14	8.04	9	8.46	0.21
10	Width between zygomatic processes	60.04	87.21	80.97	1.07	100.29	127.85	114.6	2.54	119.66	135.63	128.23	4.16
11	Maximum braincase width	41.76	53.22	50.39	0.36	60.08	68.05	63.77	0.7	60.08	71.86	66.8	2.55
12	Width behind the orbital process	19.73	33.86	29.53	0.46	36.47	41.56	38.84	0.53	39.55	41.84	40.6	0.47
13	Minimum width between orbita	19.56	29.36	25.58	0.44	31.69	44.91	37.96	1.29	40.8	45.51	43.54	1.08
14	Canine length lower	3.96	7.38	6.6	0.14	10.43	14.54	11.71	0.34	12.81	13.88	13.25	0.23
15	Canine width lower	3.42	6.03	4.85	0.1	6.48	8.65	7.32	0.19	8.04	9.01	8.54	0.26
16	Mandible: height between coronoid & angular process	29.06	49.01	42.98	0.77	51.66	68.14	61.07	1.28	62.84	75.87	68.31	3.02
17	Height between coronoid	14.7	24.15	21.42	0.36	25.77	34	30.93	0.75	26.5	36.61	32.06	2.42
18	Height of body behind m1	2.05	4.72	3.36	0.09	3.88	8.07	5.23	0.42	6.89	7.97	7.2	0.26
19	M1 length	11.05	19.36	17.7	0.25	20.78	30.02	24.24	0.69	23.99	28.01	25.8	0.84
20	Width of lower carnassial	4.14	7.65	6.97	0.11	8.38	10.28	9.21	0.19	10.25	15	12.41	1.36
S no.	Body Characters	Golden jackal (n = 52)				Indian wolf (n = 11)				Himalayan wolf (n=4)			
1	Head length	10	23.8	17.32	0.39	24.1	29.2	26.12	0.5	28.3	35.2	31.42	9.44
2	Ear height	3.3	9	6.96	0.15	9	13.5	11.2	0.44	9.55	11.9	10.41	10.14
3	Body length	33.6	68.8	53.93	1.06	72	94.5	79.95	2.26	58.6	113.5	84.79	3.69
4	Tail length	10.4	31.4	23.54	0.63	9.5	38.9	30.83	2.46	38.1	47.5	42.12	8.81
5	Fore limb length	12.1	35.9	26.29	0.66	25.6	50.5	40.46	2.48	38.55	45.1	41.7	9
6	Hind limb length	12.15	36.15	25.98	0.68	24.75	49.05	41.35	2.6	37.8	43.95	39.66	13.96

**Table 2.** Medulla and cuticular characteristics of guard hair from wild species of genus *Canis* in India.

Hair characteristics	Indian wolf (n = 10)	Himalayan wolf (n = 10)	Golden jackal (n = 10)
Cuticular characteristics			
Scale margin	Crenate	Crenate	Crenate
Scale distance	Near	Near	Near
Scale pattern	Irregular wave	Irregular wave	Irregular wave
Medulla characteristics			
Type	Simple and Wide	Simple and Wide	Simple and Wide
Medulla thickness mean (SE)	0.075 (0.0010) mm	0.054 (0.0013) mm	0.057 (0.0008) mm
Percentage medulla mean (SE)	70.2% (0.47)	81.4% (0.7)	71.1% (0.56)

## References

[B6871046] Aggarwal Ramesh, Ramadevi J, Singh Lalji (2003). Ancient origin and evolution of the Indian wolf: evidence from mitochondrial DNA typing of wolves from Trans-Himalayan region and Pennisular India. Genome Biology.

[B6870974] Bahuguna A., Sahajpal V., Goyal S., Mukherjee S. K., Thakur V. (2010). Species identification from guard hair of selected Indian mammals- a reference guide.

[B6871129] Bellis C., Ashton K. J., Freney L., Blair B., Griffiths L. R. (2003). A molecular genetic approach for forensic animal species identification. Forensic Science International.

[B6871014] Boitani L., Phillips M, Jhala Y. V. (2020). *Canislupus* (errata version published in 2020). The IUCN Red List of Threatened Species 2018: e.T3746A163508960..

[B6870990] Brunner H., Coman B. J. (1974). The identification of mammalian hairs.

[B6870901] Chakraborty R., De J. K. (2001). Identification of dorsal guard hairs of five indian species of the family Canidae (Carnivora: Mammalia). Mammalia.

[B6870871] Chawla Malaika Mathew, Srivathsa Arjun, Singh Priya, Majgaonkar Iravatee, Sharma Sushma, Punjabi Girish, Banerjee Aditya (2020). Do wildlife crimes against less charismatic species go unnoticed? A case study of golden jackal *Canisaureus* Linnaeus, 1758 poaching and trade in India. Journal of Threatened Taxa.

[B6870948] Frankham Richard, Ballou Jonathan D., Briscoe David A., McInnes Karina H. (2002). Introduction to conservation genetics.

[B6870883] Gipson Philip S., Ballard Warren B., Nowak Ronald M., Mech L. David (2000). Accuracy and precision of estimating ege of Gray wolves by tooth wear. The Journal of Wildlife Management.

[B6870956] Hinton Joseph W., Chamberlain Michael J. (2014). Morphometrics of *Canis* taxa in eastern North Carolina. Journal of Mammalogy.

[B6871006] Hoffmann M., Arnold J., Duckworth J. W., Jhala Y., Kamler J. F., Krofel M (2020). *Canisaureus* (errata version published in 2020). The IUCN Red List of Threatened Species 2018:e.T118264161A163507876. https://dx.doi.org/10.2305/IUCN.UK.2018-2.RLTS.T118264161A163507876.en. Downloaded on 19 January 2021..

[B6870892] Jhala Yadvendradev V., Giles Robert H. (1991). The status and conservation of the wolf in Gujarat and Rajasthan, India. Conservation Biology.

[B6907759] Jhala Y V., Sharma D K. (1997). Child-lifting by wolves in eastern Uttar Pradesh, India. Journal of Wildlife Research.

[B7195307] Jhala Y., Moehlman P. D., C.Sillero-Zubiri, M.Hoffmann C., W.Macdonald D. (2004). Canids: foxes, wolves, jackals and dogs. Status survey and conservation action plan.

[B6907703] Jhala Y. V. (2003). Status, ecology and conservation of the Indian wolf *Canislupuspallipes* Sykes. Journal of the Bombay Natural History Society.

[B6888580] Jolliffe I,T (2002). Principal Component Analysis.. 2nd ed. Springer-Verlag New York, Inc.493 p.

[B6870862] Kennedy Alan. J. (1982). Distinguishing characteristics of the hairs of wild and domestic canids from Alberta. Canadian Journal of Zoology.

[B6870939] Khosravi R., Kaboli M., Imani J., Nourani E. (2012). Morphometric variations of the skull in the gray wolf (*Canislupus*) in Iran. Acta Theriologica.

[B6871022] Koppikar B. R., Sabnis J. H. (1976). Identification of hairs of some Indian mammals..

[B6871064] Milenković Miroljub, Šipetić Vida Jojić, Blagojević Jelena, Tatović Svetislav, Vujošević Mladen (2010). Skull variation in Dinaric Balkan and Carpathian gray wolf populations revealed by geometric morphometric approaches. Journal of Mammalogy.

[B6907674] Nowak R. M., Federoff N. E. B. (2002). The systematic status of the Italian wolf *Canislupus*. Acta Theriologica.

[B6870853] Okarma Henryk, Buchalczyk Tadeusz (1993). Craniometrical characteristics of wolves *Canislupus* from Poland. Acta Theriologica.

[B6870921] Onar Vedat, Belli Oktay, Owen Pamela R. (2005). Morphometric examination of red fox (*Vulpesvulpes*) from the Van-Yoncatepe necropolis in Eastern Anatolia. International Journal of Morphology.

[B6871084] Pillay Rajeev, Johnsingh A. J.T., Raghunath R., Madhusudan M. D. (2011). Patterns of spatiotemporal change in large mammal distribution and abundance in the southern Western Ghats, India. Biological Conservation.

[B7195373] RCoreTeam (2013). R: A language and environment for statisticalcomputing. http://www.R-project.org/..

[B6871149] Rutledge L. Y.,, White B. N.,, Row J. R.,, Patterson B. R. (2012). Intense harvesting of eastern wolves facilitated hybridization with coyotes. Ecology and Evolution,.

[B6870965] Sari A., Arpacik A. (2018). Morphological hair identification key of common mammals in Turkey. Applied Ecology and Environmental Research.

[B6871111] Sharma D. K.,, Maldonado J. E.,, Jhala Y. V.,, Fleischer R. C. (2004). Ancient wolf lineages in India.. Proceedings of the Royal Society of London. Series B: Biological Sciences.

[B6870910] Singh G., Srinivas Y., Kumar G. C., Singh A., Sharma C. P., Gupta S. K. (2020). Identification of selected wild felids using hair morphology and forensically informative nucleotide sequencing (FINS): Wildlife forensics prospective. Legal Medicine.

[B6871055] Stoyanov Stoyan (2020). Cranial variability and differentiation among golden jackals (*Canisaureus*) in Europe, Asia minor and Africa. ZooKeys.

[B6870930] Vanak Abi Tamim, Gompper Matthew E. (2009). Dogs *Canisfamiliaris* as carnivores: Their role and function in intraguild competition. Mammal Review.

[B6871120] Vilà C., Wayne R. K. (1999). Hybridization between wolves and dogs. Conservation Biology.

[B7195320] Wayne R. K. (1986). Cranial morphology of domestic and wild canids: the influence of development on morphological change. Evolution.

[B6871038] Wozencraft W. C. (2005). Order Carnivora. In: Wilson DE, Reeder DM (eds) Mammal species of the world: a taxonomic and geographic reference.

[B6871074] Yumnam Bibek, Negi Tripti, Maldonado Jesús E., Fleischer Robert C., Jhala Yadvendradev V. (2015). Phylogeography of the golden jackal (*Canisaureus*) in India. PLoS One.

